# Prospects of antibodies targeting CD47 or CD24 in the treatment of glioblastoma

**DOI:** 10.1111/cns.13714

**Published:** 2021-08-06

**Authors:** Hao Wu, Jialin Liu, Zhifei Wang, Wen Yuan, Ling Chen

**Affiliations:** ^1^ The Third Xiangya Hospital of Central South University Changsha China; ^2^ Chinese PLA General Hospital and PLA Medical College Chinese PLA Institute of Neurosurgery Beijing China; ^3^ Zhuzhou Central Hospital Zhuzhou China

**Keywords:** anti‐CD47/CD24 antibody, cancer stem cells, glioblastoma, innate immunity, tumor‐associated macrophages

## Abstract

Glioma is a malignant tumor with the highest incidence among all brain tumors (about 46% of intracranial tumors) and is the most common primary intracranial tumor. Among them, glioblastoma (GBM) is highly malignant and is one of the three refractory tumors with the highest mortality rate in the world. The survival time from glioblastoma diagnosis to death is only 14–16 months for patients with standard treatment such as surgery plus radiotherapy and chemotherapy. Due to its high malignancy and poor prognosis, in‐depth studies have been conducted to explore effective therapeutic strategies for glioblastoma. In addition to the conventional surgery, radiotherapy, and chemotherapy, the glioblastoma treatments also include targeted therapy, immunotherapy, and electric field treatment. However, current treatment methods provide limited benefits because of the heterogeneity of glioblastoma and the complexity of the immune microenvironment within a tumor. Therefore, seeking an effective treatment plan is imperative. In particular, developing an active immunotherapy for glioblastoma has become an essential objective in the field. This article reviews the feasibility of CD47/CD24 antibody treatment, either individually or in combination, to target the tumor stem cells and the antitumor immunity in glioblastoma. The potential mechanisms underlying the antitumor effects of CD47/CD24 antibodies are also discussed.

## Graphical abstract

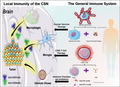

## 1 INTRODUCTION

Glioblastoma has a sophisticated immune microenvironment that is different from other solid tumors. The central nervous system was once considered an immune exemption organ because of the lack dedicated lymphatic channels and the limited presentation of antigens derived from the brain to the peripheral immune cells. In 2015, Louveau et al.^1^ defined a new lymphatic system. Most antigen‐presenting cells that leave the brain may migrate into the deep cervical lymph nodes, where they activate T and B lymphocytes. Since then, immunotherapy for glioblastoma has attracted the interest of researchers. However, systemic immunity, especially the cellular immune function, is suppressed in patients with glioblastoma or in mouse models of glioblastoma.^2^ Therefore, enhancing the patient's adaptive immunity, or turning “cold tumor” into “hot tumor,” has become the primary goal for glioblastoma research. There have been several potential treatments for glioblastoma, such as immune checkpoint inhibitors and CAR‐T. However, the enthusiasm toward immunotherapy has been dampened because not all patients with glioblastoma benefit from these treatments. Studies on the microenvironment of glioblastoma indicate that the number of microglial cells and macrophages in the tumor exceeds the infiltrating T cells.^3^ The lack of T cells in the tumor microenvironment is different from other tumor types, such as melanoma or lung cancer.^4^ Therefore, myeloid‐derived cells may be the key to glioblastoma, and controlling their differentiation and polarization may bear the same importance as activating adaptive immunity.

It is recently discovered that the tumor conveys the "don't eat me" signal of innate immune surveillance through CD47‐SIRPα^5^ and CD24‐Siglec‐10^6^ action. In preclinical studies, antibodies targeting CD47/CD24 yield encouraging results in various types of tumors.^7^, ^8^, ^9^, ^10^, ^11^ A variety of anti‐CD47 antibodies have entered clinical trials (Table 1). Further studies have found that CD47 can affect the polarization of tumor‐associated macrophages,^12^ while CD24 has no relevant reports. The M2‐type polarization of tumor‐associated macrophages can promote tumor growth, invasion, blood vessel formation, etc.^13^, ^14^ In addition, CD47/CD24 is expressed in tumor stem cells and cause the emergence of tumor resistance and promote tumor recurrence.^15^, ^16^

**TABLE 1:** Regulatory factors of tumor immunity

Tumor Immune Regulatory Factors	
Intrinsic Factors in Tumor Cells	External Factors of Tumor Cells
Signaling via mitogen‐activated protein kinases^131^	Expression of immune checkpoint molecules
Acquired mutations encoding the phosphatase PTEN^131^	Infiltration by myeloid‐derived suppressor cells^132^
Activation of the WNT–β‐catenin pathway^133^	Desmoplastic tumor stroma (a barrier to lymphocyte infiltration)^134^
Alterations signaling via the cytokine IFN‐γ^133^	
Loss of heterozygosity of loci containing genes encoding human leukocyte antigens^133^	
Downregulation of neoantigens^135^	

The primary target cells of anti‐CD47/CD24 antibodies are microglia/macrophages.^6^, ^9^ Thus, we believe that these antibodies may trigger an antitumor immune response by activating myeloid innate immune cells. When used together with immune checkpoint inhibitors that activate systemic immunity, this treatment could offer surprising effects.

## 2 GLIOBLASTOMA

Glioblastoma (GBM) is a common malignant tumor that originates in the brain. According to CBTRUS (Central Brain Tumor Registry of the United States), glioblastoma accounts for 14.9% of all brain tumors in the United States. This tumor is characterized by its prominent invasiveness and poor prognosis. The 5‐year survival rate for GBM is as low as 5.5%.^17^ At present, the standard treatment of GBM is mainly the extensive surgical resection, supplemented by radiotherapy and temozolomide chemotherapy.^18^ An array of new biomarkers for glioblastoma has been identified recently.^19^, ^20^, ^21^ The mechanisms underlying tumor cell growth and invasion have been elucidated.^22^, ^23^ New treatment strategies for glioblastoma have been under active research, including the targeted therapy based on molecular biomarkers,^24^, ^25^, ^26^ alternating electric field therapy that acts on mitosis of tumor cells,^27^, ^28^ and immunotherapy that targets different aspects of tumor immunity.^29^, ^30^, ^31^, ^32^, ^33^ Based on current research results, we need to pay attention to two key factors that affect the effectiveness of glioblastoma treatment: tumor stem cells (TSC) and tumor immune microenvironment.

### 2.1 Glioma tumor stem cells

In 2016, the World Health Organization classified glioblastoma into four different subtypes based on gene mutations and high expression of specific biomarkers: anterior, neurological, classic, and interstitial glioblastoma.^34^, ^35^ Glioblastoma is viewed as an aggressive tumor with substantial heterogeneities.^36^ Sottoriva et al. performed a complete genomic analysis on biopsies of various parts of the tumors from 11 glioblastoma patients. The results revealed extensive intratumoral heterogeneities.^37^ Among many different subtypes of glioblastoma cells are a subpopulation of tumor stem cells, which have the characteristics of stem cells and the ability to differentiate into tumor cells.^38^ Numerous studies have suggested that this relatively small subset of cells may be the driving force behind tumor recurrence.^39^, ^40^ Kamalakannan et al. used graded‐dose radiotherapy and temozolomide to isolate a subset of cells called treatment‐resistant tumor‐initiating cells (TRTICs) from xenotransplanted gliomas.^41^ They found that the cloning, self‐renewal, continuous xenotransplantation, and differentiation potential of TRTICs are surprisingly similar to tumor stem cells. Furthermore, they discovered that TRTICs could tolerate both radiotherapy and chemotherapy, and these cells are characterized by the expression of surface markers such as CD44 and CD24.^41^

**FIGURE 1:**
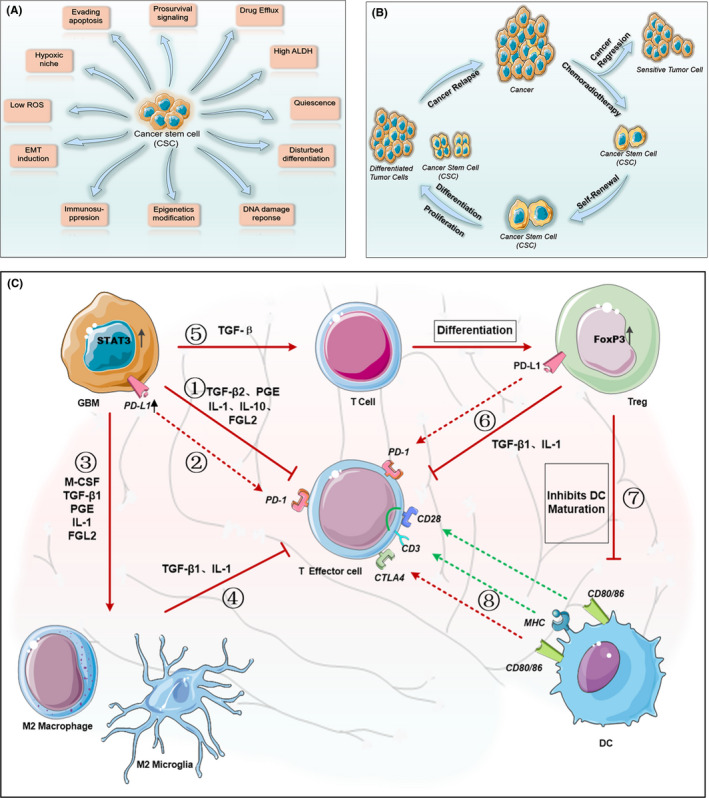
Characteristics of tumor stem cells and composition of immunosuppressive microenvironment in glioblastoma. Cancer stem cells (CSCs) are the main cause of tumor resistance and recurrence. (A) current research shows that the drug resistance mechanisms of tumor stem cells can be divided into 11 aspects. (B) After standard treatment, most of the sensitive tumor cells are cleared. However, tumor stem cells undergo self‐renewal, proliferation, and differentiation through complex mechanisms to escape host defense, and eventually lead to tumor recurrence. Therefore, targeting tumor stem cells to eradicate the tumor is a promising treatment. (C) Glioblastoma immunosuppressive microenvironment. Driven by increased expression of STAT3, glioblastoma cells (1) Secrete immunosuppressive factors such as TGF‐β2, PGE, IL‐1, IL‐10, and FGL2, which are involved in suppressing the activity of effector cells. (2) Up‐regulate PD‐L1 expression on their surface, which binds to PD‐1 on the effector cells and further inhibits effector cell activity. (3) Produce M‐CSF, TGF‐β1 and IL‐10 to polarize macrophages and microglia to immunosuppressive M2 phenotype. (4) M2 tumor‐associated macrophages secrete TGF‐β1 and IL‐10, which are also involved in suppressing effector T cells. (5) TGF‐β secreted by glioma cells causes T cells to express FoxP3 and differentiate into Treg cells. (6) Treg cells secrete TGF‐β1 and IL‐10 and expresses PD‐L1, which further suppress immunoreactive T cells. (7) Treg cells with high expression of FoxP3 inhibit the maturation of dendritic cells and hinder the effective presentation of antigen upon activation by costimulatory signals of CD80/86 and CD28. (8) The MHC molecules of dendritic cells present antigens to effector T cells and induce antigen‐specific immunity. However, CD80/86 may also inhibit the activity of effector T cells by binding to the highly expressed CTLA4 receptor. ROS: reactive oxygen species, ALDH: acetaldehyde dehydrogenase, EMT: epithelial–mesenchymal transition, TGF‐β: Transforming Growth Factor‐β, PGE: Prostaglandin E, IL: interleukin, FGL2: fibrinogen‐like protein 2, PD‐L1: programmed death ligand 1, PD‐1: programmed death 1, M‐CSF: macrophage colony‐stimulating factor, MHC: major histocompatibility complex

It is believed that the vast heterogeneities of tumor cells contribute to the current failures in treating glioblastoma in the clinic.^42^ Since these heterogeneous tumor cells may have been differentiated from TSCs,^43^, ^44^ a logical strategy for glioblastoma treatment would be targeting the TSCs. To do so, one must understand the mechanism by which TSCs escape from therapeutic targeting. An increasing body of evidence suggests that specific anti‐apoptotic and pro‐survival pathways are activated, and the drug effluxes increased in TSCs (Figure 1A,B), which all contribute to the development of glioblastoma resistance to antitumor therapies.^45^ Due to the functional differences between TSCs and their differentiated offspring cells at the transcriptome, epigenetics, and metabolic levels,^46^ no single treatment is currently effective for glioblastoma. Therefore, the development of new therapies targeting TSCs may be the key to “eradicating” the tumor.

### 2.2 Immunosuppression of glioblastoma

After the body recognizes a tumor antigen, antigen‐presenting cells present it to effector cells. The effector cells then carry out immune responses to specifically kill tumor cells. This process, which is defined as tumor immunity, is quite complex and is regulated by multiple pathways. See Table 1 for details.

To achieve effective immunotherapy for glioblastoma, one must overcome two significant obstacles: 1. immune‐privileged; and 2. immunosuppression.

It is previously believed that the central nervous system is an immune‐privileged organ lacking antigen‐presenting cells. Microglia cells present in brain tissue under noninflammatory conditions and play a limited antigen‐presenting role but are less effective than the peripheral macrophages.^47^ The expression of major histocompatibility complexes is reduced in microglia.^48^ In addition, a single‐cell sequencing research reported that tumor‐associated macrophages overexpressed genes encoding MHCII components (H2‐Aa, H2‐Ab1, ANDH2‐Eb1), as well as CD52, a costimulatory signal, which mediates T‐cell activation and proliferation. There indirectly suggested that tumor‐associated macrophages may also be involved in antigen presentation during tumor immunity.^49^ Although activated microglia can present antigens to active lymphocytes,^13^, ^50^ the presentation of tumor antigens and cross‐activation of T lymphocytes are mainly performed by dendritic cells rather than microglia.^51^ Whether microglia can perform effective antigen presentation and activate cellular immunity in the central nervous system requires further research.

Numerous studies have confirmed that patients with glioblastoma experience both systemic and local immunosuppression. Systemic immunosuppression, characterized by impaired cell‐mediated immune function,^52^, ^53^ occurs in glioblastoma patients after radiation or astemizole chemotherapy. In these patients, bone marrow T cells fail to enter the circulation.^54^ Among glioblastoma patients treated with amide, 73% exhibit significant decreases in peripheral CD4+ T cells.^55^ Immune suppression also occurs locally in the tumor microenvironment. It is regulated by glioma cells, tumor‐related macrophages, Treg cells, effector T cells, and cytokines such as IL‐1, IL‐10, TGF‐β, and FGL2^56^, ^57^ (Figure 1C).

Compared with other malignant solid tumors, glioma tissue contains many tumor‐associated macrophages (TAMs), while lacks effector T lymphocytes and dendritic cells. Such cell composition promotes tumor development and suppresses antitumor immunity.^58^

Microglia are differentiated tissue macrophages entering in the central nervous system during embryonic development.^59^, ^60^, ^61^ Some border‐associated macrophages (BAM), such as choroid plexus meninges, or perivascular macrophages,^62^ are also important CNS myeloid cells and some of them can be partially supplemented by circulating monocytes.^63^, ^64^ Like the traditional macrophages, all the above cells in the brain possess many functions, including scavenging, phagocytosis, antigen presentation, and migration.^60^ It is believed that although glioblastoma contains myeloid cells such as microglia and tumor‐associated macrophages, it usually lacks antigen‐presenting dendritic cells, and tumor‐infiltrating lymphocytes.^3^, ^65^, ^66^ Chu et al. reported that due to the upregulation of endogenous TLR2 ligand in tumor tissues, the expression of MHC II in microglia is suppressed,^67^ which ultimately hinders the antigen presentation and promotes tumor escape.

Recently, it has been suggested that tumor‐infiltrating microglia and macrophages, so‐called tumor‐associated microglia (TA‐MG) and tumor‐associated macrophages (TA‐MAC), respectively, play essential roles in shaping the microenvironment that influences glioma growth.^68^ While the classically activated pro‐inflammatory macrophages (M1) may orchestrate an antitumor immunity, the alternately activated anti‐inflammatory macrophages (M2) promotes tumor growth.^13^, ^14^, ^69^ The transformation of TA‐MG and TA‐MAC to the M1 or M2 phenotype depends on the clues provided by the local microenvironment, for example, cytokines. Many inflammation‐resolving cytokines such as TGFbeta, M‐CSF, IL‐13, IL‐4, and IL‐10 can polarize TA‐MAC or TA‐MG into the M2 phenotype, whereas cytokines including INFgamma, MCP‐1, and TNFalpha promote the M1 phenotype. Therefore, from a future clinical translation perspective, strategies that mobilize M1 microglia and macrophage to boost antitumor immunity would have therapeutic potentials.

## 3 CD47

CD47 is a transmembrane protein widely distributed on the surface of normal cells. It consists of a highly hydrophobic transmembrane region and a hydrophilic carboxyl‐terminal cytoplasmic region. Signal‐regulated protein alpha (SIRPα), thrombospondin 1 (TSP‐1), and integrin are all CD47 ligands. Under physiological conditions, CD47 mediates cell proliferation, migration, phagocytosis, apoptosis, and activation of T cells through the action of corresponding ligands.^70^, ^71^, ^72^ SIRPα is a membrane protein of the immunoglobulin superfamily and is particularly abundant in myeloid hematopoietic cells such as macrophages and dendritic cells.^73^, ^74^ CD47 binds to SIRPα expressed on macrophages, activates the Src homology 2 domain containing tyrosine phosphatase, inhibits the accumulation of myosin in the phagocytic synapse, and finally produces “don't eat me” signal.^75^, ^76^, ^77^ Therefore, CD47 plays an important role in the normal body.

### 3.1 CD47 and tumor

CD47 is involved in regulating tumor invasion and metastasis, and the underlying mechanisms have been extensively studied.^78^, ^79^ Studies have shown that CD47 is overexpressed in almost all types of tumors and tumor stem cells, including gliomas, acute myeloid leukemia, non‐Hodgkin's lymphoma, and breast cancer. This overexpression is positively correlated with poor prognosis.^5^, ^15^, ^80^, ^81^ Therefore, an anti‐CD47 antibody therapy is warranted.

**FIGURE 2:**
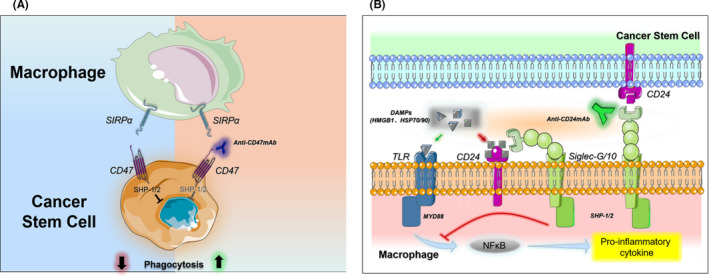
Antitumor mechanisms of CD47 and CD24 antibodies. (A) Anti‐CD47 antibodies promote macrophage phagocytosis of tumor cells by blocking the “don't eat me” signal. (B) CD24 antibody promotes tumor immune clearance and its potential adverse reactions. After blocking the highly expressed CD24 molecule on the surface of tumor stem cells, CD24 antibody prevents the activation of CD24‐Siglec‐g/10 signal, allowing macrophages to recognize tumor cells for immune clearance. In the inflammatory state, binding of TLR and DAMPs (such as HMGB1) activates the NFκB pathway, which ultimately leads to the release of pro‐inflammatory factors. The binding of CD24 and Siglec‐g/10 on the surface of macrophages to DAMPs through immune receptors tyrosine inhibitory motif (ITIM) signals blocks inflammatory process. In the presence of CD24 antibody, this inflammatory "braking" signal is seriously affected, leading to the emergence of cytokine storms. HGF/SF: hepatocyte growth factor/scatter factor, MCP‐1: monocyte chemoattractant protein‐1, M‐CSF: macrophage colony‐stimulating factor, CL3CL1: fractalkine, CXCL, CCL: Chemokine Ligand, GD3: tumor‐derived ganglioside, iGb3: endogenous antigen, MICA/B (MHC class I chain‐related molecules A/B). DAMPS: damage associated molecular patterns, HMGB1: high mobility group box 1 protein, HSP: heat shock Proteins, TLR: toll‐like receptors, MYD88: myeloid differentiation factor 88

Numerous preclinical studies indicated that the monoclonal anti‐CD47 antibody has an excellent antitumor effect. The underlying mechanism mainly includes the following: (1) CD47 antibody blocks the CD47‐SIRPα axis, thereby promoting the phagocytosis of tumor cells by macrophages (Figure 2A). This mechanism has been validated in studies of various tumors such as glioma,^8^ lymphoma,^9^ and myeloid leukemia.^9^ (2) CD47 antibody can induce tumor cell apoptosis. CD47 antibody directly induces apoptosis in cultures of several types of hematopoietic cancer cells.^7^ CD47 antibody‐induced apoptosis appears to be caspase‐independent.^82^ Morphologically, this type of apoptosis manifests typical features of apoptosis, including cell contraction and decreased mitochondrial transmembrane potential, without chromatin condensation or DNA fragmentation. (3) CD47 antibody induces cell‐mediated cytotoxicity through Fc receptors.^5^ (4) CD47 antibody promotes the antigen presenting by dendritic cells (DCs),^83^ thus improving the recruitment of T cells.^51^, ^84^ In vivo studies have shown that blocking CD47 enhances the phagocytosis of macrophages and cytoplasmic sensation of tumor cell DNA, thereby activating the innate immunity through the STING signal and activating T cells through DCs.^85^ (5) Several studies showed that CD47 could facilitate the transformation of TA‐MACs to the M1 phenotype and therefore inhibit tumor growth.^12^ In a similar mechanism, CD47 antibody can promote the phagocytosis of glioma cells in the central nervous system by activating TA‐MG.^86^ Clinical trials testing the monoclonal anti‐CD47 antibody have been carried out in a variety of solid cancers and hematopoietic cancers (see Table 2). The anti‐CD47 antibody was used either alone or in combination with other antibodies (Rituximab, Cetuximab, Nivolumab) or adjuvant therapy. However, these combination treatment trials are primarily in phase I.

**TABLE 2:** Clinical trial of tumor immunity associated with CD47 antibody

Start year	NCT ID	Cancer Types	Interventions	Individual & Combination	Format & IgG	Characteristics
2014	NCT02216409^136^	• Solid Tumor	Hu5F9‐G4	• Monotherapy	McAb & IgG4	Phase 1
2015	NCT02678338^137^	• Acute Myeloid Leukemia	Hu5F9‐G4	• Monotherapy	McAb & IgG4	Phase 1
2015	NCT02367196^138^	• Hematologic Neoplasms	CC−90002	• Monotherapy • Combine with Rituximab	McAb & IgG4	Phase 1
2016	NCT02953782^139^	• Colorectal Neoplasms • Solid Tumors	Hu5F9‐G4	•Monotherapy •Combine with Cetuximab	McAb & IgG4	Phase 1 Phase 2
2016	NCT02663518^140^	• Hematologic Malignancies • Solid Tumor	TTI−621	• Monotherapy • Combine with Rituximab • Combine with Nivolumab	CD47 infusion protein & IgG1	Phase 1
2016	NCT02641002^141^	• Leukemia, Myeloid, Acute	CC−90002	• Monotherapy	McAb & IgG4	Phase 1
2016	NCT02890368^142^	• Solid Tumors • Melanoma • Merkel‐cell Carcinoma • Squamous Cell Carcinoma • Breast Carcinoma • Human Papillomavirus‐Related Malignant Neoplasm • Soft Tissue Sarcoma	TTI−621	• Monotherapy • Combine with PD−1/PD‐L1 Inhibitor • Combine with PEGylated interferon−2a • Combine with T‐Vec • Combine with radiation	CD47 infusion protein & IgG1	Phase 1
2016	NCT02953509^143^	• Lymphoma, Non‐Hodgkin • Lymphoma, Large B Cell, Diffuse • Indolent Lymphoma	Hu5F9‐G4	• Monotherapy • Combine with Rituximab	McAb &IgG4	Phase 1 Phase 2
2016	NCT03530683^144^	• Lymphoma • Myeloma	TTI−622	• Monotherapy • Combine with Rituximab • Combine with Nivolumab • Combine with PD−1 Inhibitor • Combine with	CD47 infusion protein & IgG4	Phase 1

Start Year	NCT Number	Conditions	Interventions	Individual & Combination	Format & IgG	Characteristics
				Proteasome‐inhibitor Regimen		
2017	NCT03248479^145^	• Acute Myeloid Leukemia	Hu5F9‐G4	• Monotherapy	McAb & IgG4	Phase 1
2018	NCT03717103^146^	• Advanced Malignancies	IBI188	• Monotherapy • Combine with Rituximab	McAb & IgG4	Phase 1
2018	NCT03512340^147^	• Advanced Solid Cancers • Hematologic Cancers	SRF231	• Monotherapy	Anti‐CD47 body & Unknown	Phase 1
2018	NCT03527147^148^	• Non‐Hodgkin's Lymphoma • Diffuse Large B Cell Lymphoma	Hu5F9‐G4	•Monotherapy	McAb & IgG4	Phase 1
2019	NCT03763149^149^	• Advanced Malignancies	IBI188	• Monotherapy	McAb & Ig G4	Phase 1
2019	NCT04097769^150^	• Advanced Solid Tumor	HX009	• Monotherapy	anti‐PD−1/CD47 infusion protein	Phase 1
2019	NCT03957096^151^	• Soft Tissue Sarcoma • Colorectal Cancer • Head and Neck Squamous Cell Carcinoma • Non‐small‐cell Lung Carcinoma • Breast Carcinoma • Ovarian Carcinoma • Exocrine Pancreatic Carcinoma • Gastric Carcinoma • Melanoma	SGN‐CD47 M	• Monotherapy	McAb& Unknown	Phase 1
2020	NCT04257617^152^	• Locally Advanced Solid Tumor	ZL1201	• Monotherapy	McAb & Unknown	Phase 1

Note

All data were obtained from www.clinicaltrials.gov. McAb: monoclonal antibody, T‐Vec: talimogene laherparepvec.

### 3.2 CD47 and the treatment of glioblastoma

At present, the application of CD47 antibodies glioblastoma remains at the preclinical stage. The main reasons for the delayed testing of CD47 antibodies in clinical gliomas are related to the concerns about the antibody itself as described below and the possible resistance of gliomas to such therapy.

### 3.3 CD47 antibody: concerns about safety and reliability

First, the off‐target effect of the CD47 antibody includes the blockage of CD47 signaling in normal erythrocytes, thus increasing the expression of calreticulin, an “eating me” signal.^87^ Meanwhile, the Fc‐mediated killing of target cells is activated, making natural killer cells (NK) attack red blood cells and cause anemia. Second, the binding of antibodies to T lymphocytes may cause T cell apoptosis and immunosuppression.^88^ Finally, the interaction between the antibody Fc fragment and the Fc receptor on macrophages plays a critical role in activating macrophages. Therefore, it is not enough to activate macrophages only by blocking CD47‐SIRPα. Taking these facts into consideration, IgG1 should be selected to make the antibody function effectively (such as TTI‐621, the effect of Ig G4 that has on activating macrophages is weaker than IgG1), which, however, would inevitably lead to attacking on RBCs and T lymphocytes by immune cells. In order to avoid cytotoxicity, IgG4 has been selected in most studies—that is, to obtain the safety at the expense of effectiveness (such as CC‐90002, Hu5F9‐G4, and IBI188), consequently, the expected result can only be achieved when other antibody drugs with ADCC/ADCP activities are combined.

### 3.4 Treatment resistance: Immune privilege and immunosuppression

Compared with other organs, brain parenchyma is separated from blood circulation by the blood‐brain barrier. The lack of professional antigen‐presenting cells in the brain makes antigen recognition and presenting difficult. Although CD47 antibodies can promote the conversion of TA‐MG and TA‐MAC to the M1 phenotype^12^ and upregulate the expression of MHC‐II,^67^ a marker of antigen‐presenting function, the activated microglia, or macrophages may not be enough to boost the antitumor cellular immunity. A study on colon cancer and lymphoma pointed out that the therapeutic effect of CD47 blockade on immunocompetent mice relies on dendritic cells rather than the cross‐initiation of macrophage responses to T cells.^51^

Immunosuppression is prevalent in patients with glioblastoma. The brain homing of T lymphocytes leads to a decrease in peripheral T lymphocytes.^54^ Glioblastoma cells secrete anti‐inflammatory factors such as TGFβ, PGE, IL‐1, IL‐10, and FGL2, which suppress the effector cells. Myeloid suppressor cells in the local microenvironment recruit and differentiate Regulatory T cells (Treg), and restrict DC activation^89^, ^90^ (Figure 1C). Since single immunotherapy cannot target many pathways, it is reasonable that the development of clinical immunotherapy for glioblastoma, including the use of CD47 antibodies, has progressed slowly.

Considering these reasons, it is necessary to combine CD47 antibody with other anti‐GBM therapies to increase efficacy while reducing drug resistance. A recent report revealed that administration of RRx‐001, an anti‐CD47‐SIRPα small molecule with vascular normalizing properties, prior to temozolomide or irinotecan results in increased drug uptake in orthotropic glioma tumors.^91^ In terms of combination of immunotherapy, the CD47 antibody that focuses on re‐education of TA‐MG and TA‐MAC needs to be combined with other immunotherapy such as dendritic vaccines and adoptive T‐cell therapy to treat gliomas. Future glioblastoma immunotherapy should aim to promote efficient innate immunity, improve antigen‐presenting efficiency, enhance cellular immune activity, and relieve tumor immune tolerance and immunosuppression.

## 4 CD24

CD24 is a protein anchored to the cell membrane by glycosylphosphatidylinositol (GPI). CD24 proteins from different tissues or cell types possess different molecular weights (ranging from 20 to 70 kD^92^, ^93^, ^94^, ^95^). Because the glycosylation of CD24 is highly variable and cell type‐specific, it binds to different cell ligands to perform various functions. For example, in the brain, CD171, Tag‐1, and contactin can bind to CD24 and induce nerve growth inhibition.^96^, ^97^, ^98^ In hematopoietic cells, CD24 binds to danger‐associated molecular pattern (DAMP) molecules and sialic acid‐binding immunoglobulin‐like lectins to form a three‐molecule complex, thereby blocking Toll‐like receptor (TLR)‐mediated inflammation and macrophage phagocytosis^99^ (Figure 2B). As a GPI anchor molecule, it also recruits Src family protein tyrosine kinase (Ptk) through membrane rafts to mediate signal transduction and participate in the development and apoptosis of B cells and T cells, cell binding, and granulocyte oxidative burst.^100^, ^101^, ^102^, ^103^, ^104^, ^105^, ^106^, ^107^

### 4.1 CD24 and tumor

CD24 is widely expressed on various hematopoietic cells^108^, ^109^, ^110^ and nonhematopoietic cells.^94^, ^111^, ^112^, ^113^ A review by Fang et al. suggests that CD24 is expressed at higher levels in progenitor cells or metabolically active cells but lower levels in terminally differentiated cells.^114^ CD24 showed substantially high expression levels in various cancers and cancer stem cells, such as breast cancer,^115^, ^116^ pancreatic cancer,^117^ and glioma.^118^ CD24 overexpression is positively correlated with the pathological grade or prognosis of cancer. This information is of great significance for targeting cancer stem cells and formulating appropriate treatment plans for rapidly proliferating cancers.

Lipid rafts are cholesterol‐rich environments in cell membranes and participate in cell signal transduction. CD24 is linked to various signaling pathways through GPI. Simultaneously, through highly variable glycosylation, CD24 also affects the growth of tumors (Table 3). Studies have shown that CD24 promotes cancer cell adhesion,^119^ growth,^116^ proliferation,^120^ invasion,^121^ and metastasis,^116^ while inhibits cancer cell apoptosis.^122^ In addition, CD24 has been proposed as a biomarker for the active proliferation of several types of cancer stem cells. Therefore, CD24 has attracted much attention as a potential molecule to target cancer cells/cancer stem cells.

**TABLE 3:** Mechanisms of CD24 involved in tumorigenesis due to glycosylation characteristics and different binding ligands

The Role of CD24 in Cancer Development			
Glycosylation Mediate Mechanisms		Ligand Mediated Mechanisms	
Sialyl‐Lewis (x) promotes	Metastasis^153^	P‐selectin	Metastasis^153^
N‐acetylglucosamine	CSCs Self‐renewal and tumorigenicity^154^	E‐selectin (CD62E)	Transfer and Scrolling^155^
		L1 (CD171 or L1CAM)	Progress and Proliferation^156^
		Siglec‐G (mice) or Siglec−10 (humans)	Immune Evasion^99^, ^157^

Abbreviation: L1CAM, L1 Cell Adhesion Molecule.

Although CD24 has been extensively studied for its role in tumor growth, there are few reports identify CD24 as a therapeutic target. Klapdor et al. designed the third‐generation chimeric antigen receptor (CAR) for CD24, which demonstrates a high degree of cytotoxic effect on ovarian cancer cells.^123^ CD24 monoclonal antibodies have been reported to inhibit tumor growth and prolong the overall survival in the mouse models of metastatic cancers.^10^, ^11^ The most important discovery is by Amira and colleagues,^6^ who revealed that CD24 is functionally complementary to CD47 and programmed cell death ligand 1 (PD‐L1). As an important “don't eat me” signaling molecule, CD24 binds to Siglec‐G (mice) or Siglec‐10 (humans) in cancer cells, triggering the immune escape reactions. Thus, blockage of the CD24 effect by its monoclonal antibodies effectively enhances the ability of TA‐MACs to attack various types of cancer cells.^6^ Finally, the combined blockage of both CD24 and CD47 confers an additive phagocytosis‐mediated cancer‐killing effect.^6^ Thus far, anticancer therapy using the anti‐CD24 antibodies has not been tested in clinical trials, and its potential adverse effects are largely unknown. Although the mouse erythrocytes express CD24a, it does not appear that the anti‐CD24 monoclonal antibodies interact with or kill human erythrocytes.^6^

### 4.2 CD24 and the treatment of glioblastoma

The expression of CD24 is up‐regulated in glioblastoma stem cells and functionally involved in the migration, infiltration, and metastasis of glioblastoma cells.^41^, ^124^ An overexpression of CD24 by more than two fold has been associated with poor overall survival in GBM, the poor survival may be related to increased “stemness” of tumor cells,^16^ which provides a potential therapeutic target for glioblastoma. From the translation perspective, the combined application of CD24 antibody with the CD47 antibody offers an additive effect against glioblastoma compared to either treatment alone. Further clinical evaluation on this combined treatment on glioblastoma is warranted.

The mechanism by which the anti‐CD24 antibody promotes M1 polarization of TA‐MAC and TA‐MG should be further investigated. Through M1 conversion, the anti‐CD24 antibody may fundamentally alter the microenvironment that otherwise supports tumor growth. In addition, CD24 serves as an innate immune checkpoint. It is still unknown whether CD24 blockade in vivo can effectively bridge innate immunity and adaptive immunity, thereby enhancing the immune clearance of cancer cells. Protein complex formation involving DAMPs and Siglect‐10 (human) or Siglec‐G (mouse) helps avoid excessive immune responses, thus maintaining an immune homeostasis by binding to CD24.^99^ Another question is whether CD24 blockage with the anti‐CD24 antibody leads to the over‐activation of TLR‐mediated pro‐inflammatory reactions, which in turn triggers a cytokine storm, attacks normal cells, and results in autoimmune diseases. The latter is a particularly relevant concern for glioma therapy, as CD24 is widely expressed in brain parenchyma cells. The immunotherapy resistance of glioblastoma to CD24 antibody treatment needs to be further investigated (Figure 2B).

### 4.3 Perspectives

Immunotherapy has been developed as a novel treatment for glioblastoma. Such treatment needs the participation of the systemic immune system that orchestrates a strong and persistent cytotoxic response to brain glioma. However, due to the unique immune microenvironment within the central nervous system and in the glioblastoma, the development of clinical feasible immunotherapy for glioblastoma has been relatively slow.

**FIGURE 3:**
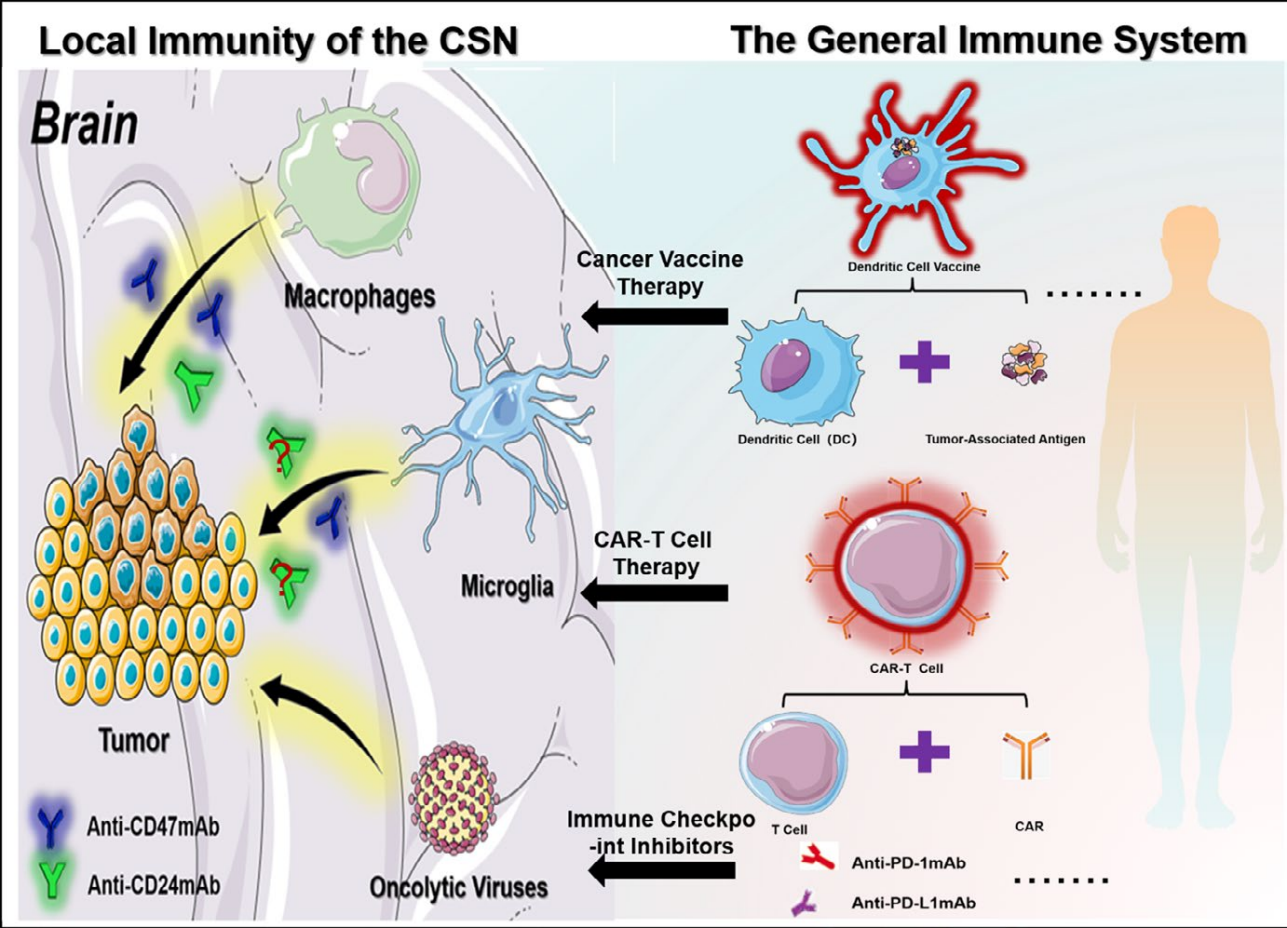
Conception of combining local immunotherapy with systemic immunotherapy. In the central nervous system, anti‐CD47/CD24 antibodies promote the phagocytosis of tumor cells by macrophages and microglia. Activating the peripheral immune system by adaptive immune system cancer vaccines (such as dendritic cell vaccines), genetically modified CAR‐T, and various immune checkpoint inhibitors (such as PD‐1, PD‐L1 antibodies) plays a synergistic antitumor therapeutic effect. This combined immunotherapy may reduce the occurrence of immune‐related adverse reactions while synergizing the antitumor effects

With the increased understanding of the mechanisms underlying cancer immunology in the glioblastoma, especially the role of the “don't eat me” CD47‐SIRPα signaling and CD24‐Siglec10 signaling, we propose here that the combined blockage of these two pathways may effectively activate the innate immune responses toward glioblastoma. CD47 and CD24 are highly expressed in cancer stem cells. The combination of anti‐CD47 and anti‐CD24 antibodies could offer robust cancer cell‐killing effects and block the vicious cycle for tumor recurrence from cancer stem cells. Previous studies have demonstrated that the anti‐CD47/anti‐CD24 dual‐antibody approach could effectively activate the myeloid immunity in the brain. We further propose that the local application of the anti‐CD47/anti‐CD24 antibodies, combined with CAR‐T, tumor vaccines, immune checkpoint inhibitors, or other systemic immunotherapies will likely improve the overall efficacy for clinical treatment of glioblastoma (Figure 3). This may represent an emerging new strategy to treat glioblastoma.

Some recent studies have suggested that the levels of CD47 and CD24 expression are positively correlated with increased angiogenesis in solid tumors,^125^, ^126^ respectively. Hence, blockage of CD47 signaling could potentiate the therapeutic effects of anti‐angiogenic therapy in certain cancer.^127^ Whether CD47 or CD24 is directly involved in angiogenesis within glioblastoma is unknown, this should be investigated in future studies. Nevertheless, given the importance of microenvironment, especially angiogenesis, in the prognosis of glioblastoma treatment, future immunotherapy against CD47 or CD24 should include the quantitative evaluations of hemodynamic changes within and surrounding the glioblastoma. Several imaging modalities have been developed,^128^, ^129^, ^130^ which can be applied to future studies as both an outcome endpoint and a potential biomarker for prognosis of immunotherapy against glioblastoma.

## CONFLICTS OF INTEREST

The authors declare no conflict of interest.

## AUTHOR CONTRIBUTIONS

Hao Wu, Jialin Liu, Wen Yuan, and Zhifei Wang were responsible for literature review and investigation; Hao Wu and Jialin Liu were responsible for draft preparation; Wen Yuan was responsible for drawing; Ling Chen was responsible for manuscript review and editing. All authors have read and agreed to the published version of manuscript.

## Data Availability

Data sharing is not applicable to this article as no new data were created or analyzed in this study.
